# The Use of a Stem and Leaf Aqueous Extract of *Cissus quadrangularis* (CQR-300) to Reduce Body Fat and Other Components of Metabolic Syndrome in Overweight Participants

**DOI:** 10.1089/acm.2018.0016

**Published:** 2019-01-18

**Authors:** Robert Nash, Boris Azantsa, Dieudonne Kuate, Harrinder Singh, Julius Oben

**Affiliations:** ^1^ PhytoQuest Limited, Plas Gogerddan, Aberystwyth, United Kingdom.; ^2^ Department of Biochemistry, University of Yaounde 1, Yaounde, Cameroon.; ^3^ Faculty of Science, University of Dschang, Dschang, Cameroon.; ^4^ Harinder Medicare & Solutions Pvt Ltd, Delhi, India.

**Keywords:** *Cissus quadrangularis*, CQR-300, impedance, DEXA, metabolic syndrome

## Abstract

***Background:*** Previous work had shown the ability of an aqueous leaf and stem extract of *Cissus quandrangularis* (300 mg of CQR, CQR-300) to improve components of metabolic syndrome (MS) in overweight individuals.

***Objective:*** This small pilot study aimed to confirm the efficacy of CQR-300 in reducing the percentage body fat measured using two different methods—bioelectrical impedance assay versus dual-energy X-ray absorptiometry (DEXA).

***Design:*** The study was an 8-week double-blind, placebo-controlled pilot trial on 67 individuals who were requested by a dietary counselor to maintain their normal exercise and dietary routines. Participants were randomly divided into two groups, placebo (32 participants) and the CQR-300 group (35 participants), and received 300 mg of corn starch or CQR-300 daily.

***Methods:*** Body fat was measured by bioelectrical impedance using a TANITA impedance meter and by DEXA, with blood samples taken at baseline and at 8 weeks for the measurement of lipid parameters.

***Results:*** After 8 weeks of treatment, participants of the placebo group showed a 1.05% decrease in body fat as determined by bioelectrical impedance analysis, but no difference using DEXA. In the same time period, the CQR-300 group had an 8.9% and 12.8% decreases in the body fat as measured by impedance and DEXA, respectively. These values were significantly (*p* < 0.05) lower than the placebo. Compared with the placebo, the CQR-300 group demonstrated significant (*p* < 0.05) decreases in the waist and hip circumferences, systolic and diastolic blood pressures, total cholesterol, triglycerides, fasting blood glucose, as well as leptin levels. On the contrary, there were significant (*p* < 0.05) increases in HDL-cholesterol and adiponectin levels.

***Conclusion:*** *CQR-300* administered as a single 300 mg dose daily was effective in reducing body fat as well as improving blood parameters associated with MS.

## Introduction

Overweight and obesity are pathologic conditions, in which excess body fat accumulates mainly in the adipose tissue to the extent that it may have adverse effects on health, leading to reduced life expectancy.^1^ On a global scale, 1.9 billion adults aged 18 years and older were overweight in 2016, with over 650 million of these being obese.^2^ This usual gradual accumulation of energy in the adipose tissue is generally accompanied by an increase in the weight of the individual, resulting from an imbalance between energy intake and expenditure. Overweight and obese individuals exhibit perturbed energy and lipid metabolism, characterized by elevated blood levels of glucose, triglycerides, and low-density lipoproteins.^3,4^ Their condition is associated with an increased oxidative damage to cellular constituents (proteins, lipids, and DNA) and increased inflammation as indicated by elevated levels of tumor necrosis factor (TNF), interleukin-1β, and other proinflammatory cytokines, which predispose to several major age-related diseases, including diabetes, cardiovascular disease, and possibly cognitive impairment, and Alzheimer's disease.^5–7^ Lifestyle changes, such as regular physical activity and nutrition improvement, are the basis for successful long-term weight loss and control of overweight and obesity.^8^ Among dietary changes, indirect evidence from a number of epidemiologic studies suggests a beneficial role of foods such as beans, vegetables, and fruits.^9,10^ The role of antioxidants in reducing the risk of metabolic syndrome (MS) has also been reported**.**^11^

Despite research efforts to curb the incidence of obesity, there is no magic pill that collectively treats all the related disorders. Thus, there is the continuous search for novel and improved therapies to manage the various components of MS.

One such therapy that has been previously reported is an aqueous extract of the leaves and stems of *Cissus quadrangularis* Linn. *C. quadrangularis* (CQR-300) is a succulent plant of the Vitaceae family commonly found in tropical and subtropical woods. It is cactus-like, of the grape family, and commonly called veld grape, devil's backbone, adamant creeper, asthisamharaka, hadjod, pirandai, and patah tulang in different societies. In India, it is a common food item as well as widely used in traditional medicine. It has been reported to possess bone fracture healing, antibacterial, antifungal, antioxidant, anthelmintic, antihemorrhoidal, and analgesic activities.^12^ *C. quadrangularis* (CQR-300) contains a variety of components, including antioxidants, indanes, as well as novel flavonoids and stilbenes with known antiobesity activity.^13^ In Sprague–Dawley rats, CQR-300 is not genotoxic nor has any observed adverse effect at a dose of 2500 mg/kg bw/day.^14^

The ability of CQR-300 to bring about weight loss and fat reduction (measured by bioelectrical impedance analysis, BIA) in obese participants has been demonstrated in several studies.^15–17^ This can be linked to its ability to inhibit key enzymes of the digestive system such as pancreatic lipase, alpha-amylase, as well as alpha-glucosidase, thereby decreasing the potential absorption of monomers.^18^ More recently, evidence from animal models of fat accumulation demonstrated that CQR-300 administration results in low expression levels of adipogenesis/lipogenesis-related genes and proteins such as C/EBPα, PPARγ, SREBP-1c, and FAS in WAT.^19^ Furthermore, phosphor-AMPK was shown to increase with CQR-300 treatment.^20^ The resulting increased clearance of LDL and VLDL, then accounts for the reduction in plasma LDL and triglyceride levels.^21^

The use of BIA to measure fat accumulation and distribution in epidemiologic and clinical studies is becoming controversial due to its inapplicability n all circumstances. Comparative studies between BIA and dual-energy X-ray absorptiometry (DEXA) seem to prove that BIA provides a relatively accurate prediction of% body fat in individuals with normal weight, overweight, or obesity following weight-loss programs, but is less accurate in predicting body fat in obese individuals at baseline or weight change during the weight-loss intervention program.^22,23^ On the contrary, an imaging technique such as DEXA is considered as the gold standard. There is a reported gender-dependent underestimation of body fat percentage measured by BIA compared to DEXA.^24^ DEXA is therefore more reliable for clinical patient follow-up, even though BIA is more versatile and has wider applications in epidemiologic studies.^25^

To ensure an accurate interpretation of the effects of administering an oral daily dose of 300 mg of CQR to overweight participants, this pilot study investigated its effect on weight and biomarkers of MS as well as compared two methods (BIA vs. DEXA) of body fat determination.

## Materials and Methods

### Plant material

*C. quadrangularis* was harvested and authenticated at the National Herbarium of Cameroon in Yaounde with the Voucher Specimen number 36966 HNC. The *C. quadrangularis* extract powder CQR-300 was prepared as previously described.^14^ In brief, the stems and leaves were washed, dried, and pulverized. This was followed by a hot water extraction and successive filtrations, before spray drying and further sieving using a 40/80 mesh. The resulting CQR-300 was validated for identity using HPTLC testing and tested for heavy metals (Pb, Hg, Cd, As) as well as a full spectrum of pesticides. The CQR-300 capsules that were used in the study contained 300 mg CQR-300 and were supplied by Gateway Health Alliances (Fairfield, CA, USA).

### Participants and methods

A total of 86 overweight subjects (BMI 25–29.9 kg/m^2^) aged between 25 and 60 years were selected from a group responding to a radio and local newspaper advertisement in Yaoundé, Cameroon. Respondents with BMI less than 25 or greater than 30 were excluded from the study. This number of participants was considered as appropriate since this was a pilot study. After physical examination and laboratory screening tests, diabetics, pregnant, and lactating women were excluded. None of these patients took any weight reducing drugs and none was following any specific diet. The purpose, nature, and potential risks of the study were explained to all patients and written informed consent was obtained before their participation. Participants were also requested not to change their habitual physical activity and food intake patterns.

### Ethical consideration

The study protocol was approved by the National Ethics Committee of Research for Human Health of Cameroon (N° 2014/08/488/CE/CNERSH/SP) and carried out according to “Guideline for Good Clinical Practice by International Conference on Harmonization,” ICH GCP. All participants voluntarily signed a consent form before the start of the intervention.

### Study design

The 8-week pilot intervention was designed as a randomized, double blind, placebo-controlled study. Participants were regularly seen by a physician and two trained attending nurses, who oversaw dispensing of the test capsules during participant visits. Randomization was done using a computer generated random number method allocating participants to one of two groups. The placebo capsules (corn starch) as well as those of the CQR-300 were identical in shape, color, and appearance. Participants received a 300 mg capsule immediately before their meal (8–10 am) and were examined every week during routine visits when their body weight, body fat, and waist and hip circumferences were measured by the attending nurse. Subjective findings such as increased or decreased appetite, feeling of lightness, and gastrointestinal pains were individually solicited and noted. Side effects of the formulation if any were noted. The participants were also interviewed about their physical activity and food intake during the study period. They were asked to keep a record of their food intake over 7 consecutive days (using household measurements). At the start of the study as well as on weeks 4 and 8, blood was collected, and plasma was prepared and stored at −70°C for between 2 and 5 days.

Allergen-specific IgE tests were performed on participants after 2 weeks of the study. The pulse rate and the respiratory rate of participants were measured at each visit.

#### Anthropometric measurements

Anthropometric measurements were done weekly. Body weight and body fat were measured using a TANITA Monitor Scale, after an overnight fast and with participants wearing light clothing. Body fat was additionally measured by fan-beam DEXA. The DEXA operator performed a whole-body scan on each participant as he/she lay down in a supine position. Whole-body composition analysis provided data on the trunk, arms, and legs. Equipment was calibrated each day by using a standardized phantom. The DEXA variable used in the present study was %BFdxa that is the percent of body fat measured by DEXA as a function of the total body weight (fat mass × 100/body weight)

Waist and hip circumferences were measured using soft nonstretchable plastic tape on the narrowest and the widest parts of the trunk.

### Blood pressure

Blood pressure was measured on participants sitting down. This was done by the attending nurse during each visit using a mercury sphygmomanometer with the cuff worn 2–3 cm above the antecubital fossa of the nondominant arm. On each occasion, three measurements were made and the average recorded.

### Sample collection and treatment

Fasting venous blood (5 mL) was collected from participants into heparinized tubes. Following centrifugation at 4500 *g* for 10 min at 4°C, plasma was collected and stored for between 2 and 5 days at −70°C before analyses.

#### Biochemical analyses of plasma

Total cholesterol in plasma was determined using an enzymatic method, while plasma triglyceride was determined as previously described.^26,27^ HDL cholesterol was determined using a heparin manganese precipitation of Apo B-containing lipoproteins, while LDL cholesterol was estimated using the Friedewald's formula.^28^ Blood glucose was determined using the glucose oxidase method.^29^ Serum leptin and adiponectin were measured using enzyme-linked immunosorbent assay.

### Statistical analysis

Results are expressed as means ± SEM. The software SPSS 20.0 was used for data analysis. Unpaired Student's *t* test was used to compare intergroup differences between the placebo and active formulation. Paired Student's *t* test was carried out on the start and end values to compare intragroup variables.

## Flow Diagram

**FIG. 1.**
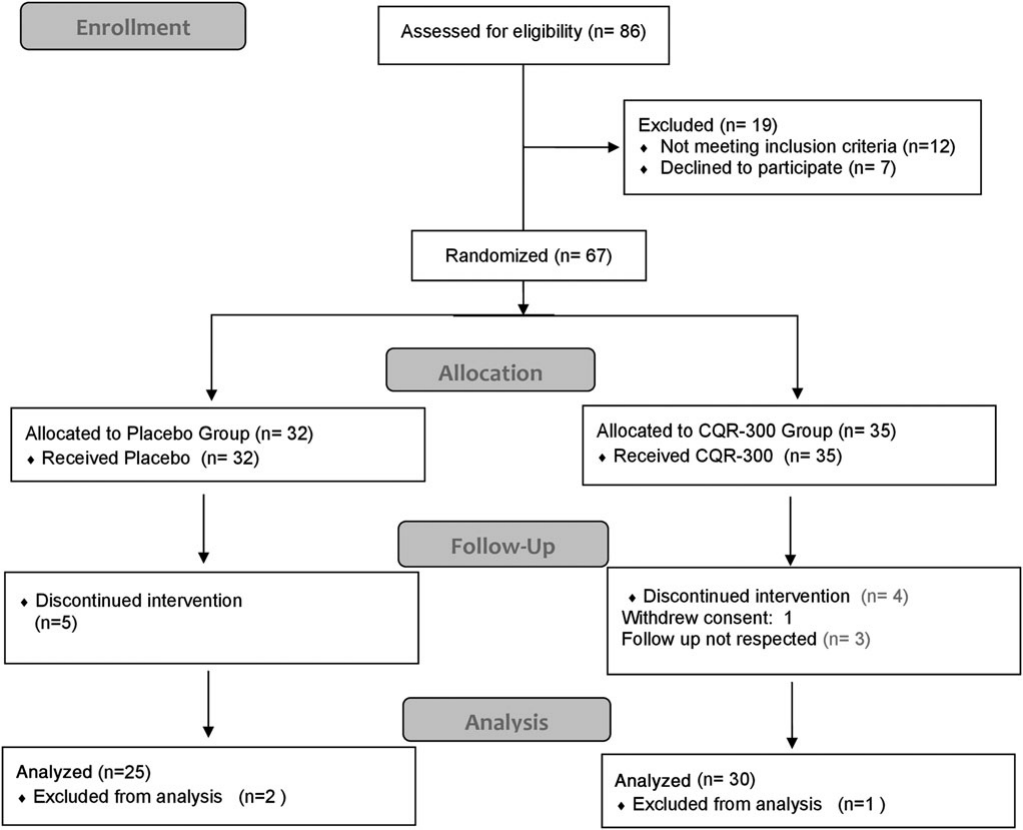
Flow diagram of study participants. CQR-300, 300 mg of *Cissus quandrangularis*.

The detailed treatment procedure is outlined in the flowchart in Figure 1.

## Results

Sixty-seven overweight individuals were included in the study. With regard to reported physical activity and food intake, no difference was observed among the participants during the 8-week study period. Participant compliance rate was over 90%. No adverse effect was reported in the CQR-300 group. Of the nine participants (five placebos and four CQR-300) who dropped out of the study, three did not think they were having any benefit, four were unable to follow the protocol, while two had malaria (endemic in study region).

### Baseline data

There were no statistically significant differences between groups at baseline for all the parameters that were measured (Table 1a).

**Table 1a.** Baseline Data

*Variable*	*Placebo* N* = 25*	*CQR-300* N* = 30*
Age	43.60 ± 8.66	43.36 ± 9.58
Sex	11 Males	16 Males
	14 Females	14 Females
Weight	92.24 ± 5.1	93.62 ± 5.63
BMI	27.66 ± 10.4	28.36 ± 7.6
Fat (impedance)	43.3 ± 8.5	43.5 ± 3.2
Fat (DEXA)	44.1 ± 8	45.3 ± 7.12
Waist circumference (cm)	94.5 ± 8	95.8 ± 7.6
Hip circumference (cm)	117.6 ± 5.5	118.5 ± 5.2
WHR	0.80 ± 0.4	0.81 ± 0.3
Systolic blood pressure (mmHg)	127.6 ± 5.5	133.2 ± 4.38
Diastolic blood pressure (mmHg)	98.2 ± 6.5	96.4 ± 6.02
Total cholesterol (mg/dL)	197.52 ± 5.1	195.7 ± 5.1
Triglycerides (mg/dL)	137.43 ± 5.35	139.13 ± 5.9
LDL cholesterol	127.61 ± 8.26	127.21 ± 8.46
HDL cholesterol (mg/dL)	42.42 ± 5.25	40.73 ± 8.90
Fasting blood glucose (mmol/dL)	4.76 ± 1.5	4.59 ± 1.31
Leptin (mg/dL)	28.78 ± 5.63	27.38 ± 12.65
Adiponectin (mcg/mL)	16.64 ± 7.75	18.34 ± 9.02

Values are means ± SD.

CQR-300, 300 mg of *Cissus quandrangularis*; DEXA, dual-energy X-ray absorptiometry.

### Adverse effects, allergies, and safety considerations

With the exception of three participants who reported being uneasy after the DEXA measurement, adverse conditions were not reported by participants nor were there any reported discomfort after administration of either the placebo or the CRQ-300 over the 8-week period. Allergen-specific IgE tests on participants after 2 weeks of the study did not indicate any signs of allergy. The pulse rate of participants did not change during the study, and ranged from 52 to 81, while the respiratory rate ranged from 15 to 22.

### Effect of CQR300 on anthropometric parameters

#### Body weight

After 8 weeks of the study, we observed weight loss in participants who received CQR-300 as well as those on placebo (Table 1b). The mean weight of participants in the placebo group dropped by 0.57 kg, while those on the CQR-300 group dropped by 7.40 kg.

**Table 1b.** Effect of CQR-300 of Weight (kg)

	*Weight T0 (Kg)*	*Weight T2 (Kg)*	*Weight T4 (Kg)*	*Weight T8 (Kg)*	*T0–T2*	*T0–T4*	*T0–T8*	*% change after 8 weeks*
Placebo	92.24 ± 5.1	92.67 ± 5.5	91.02 ± 5.2	91.67 ± 5.25	−0.43 ± 1.1	1.21 ± 2.2	0.57 ± 2.2	0.58 ± 2.3
CQR-300	93.62 ± 5.6	91.15 ± 5.3^a^	89.26 ± 6.6^*a^	86.21 ± 6.8^*a^	2.46 ± 4.0^*^	4.35 ± 2.1^*^	7.40 ± 2.5^*^	7.99 ± 3.0^*^

Values are means ± SD. Statistical significance is at ^*^p ≤ 0.05, for comparison of differences between placebo and the CQR-300 group at each time point or ^a^*p* ≤ 0.05, for comparing of differences compared to T_0_ within the same group.

CQR-300, 300 mg of *Cissus quandrangularis*.

#### Body fat

There was no significant difference in body fat measured by BIA and DEXA at baseline (T0) between placebo and CQR-300. Body fat changes as measured by bioelectrical impedance and DEXA followed a similar pattern to the changes in weight over the 8-week experimental period. Follow-up of body fatness measured by BIA showed a lot of fluctuations in percentages. Administration of CQR-300 brought a reduction of 8.9% in the treated group compared with 1.1% in the placebo group (Table 2a).

**Table 2a.** Effect of CQR-300 on Percentage Body Fat Measured by Impedance (BIA)

	*T0 (%)*	*T2 (%)*	*T4 (%)*	*T8 (%)*	*T0–T2*	*T0–T4*	*T0–T8*	*% change after 8 weeks*
Placebo	43.3 ± 8.5	42.5 ± 4	42.7 ± 4	42.8 ± 4	0.8 ± 1.0	0.6 ± 2.0	0.5 ± 2.0	1.1 ± 4.2
CQR-300	43.5 ± 0.6	43.4 ± 3.2	41.6 ± 3.8^a^	39.6 ± 4.9^*a^	0.1 ± 0.4	1.9 ± 1.6^*^	3.9 ± 2.1^*^	8.9 ± 5.0^*^

Values are means ± SD. Statistical significance is at ^*^p ≤ 0.05, for comparison of differences between placebo and the CQR-300 group at each time point or ^a^*p* ≤ 0.05, for comparing of differences compared to T_0_ within the same group.

BIA, bioelectrical impedance analysis; CQR-300, 300 mg of *Cissus quandrangularis*.

In the placebo group, body fat measured with DEXA, slightly but not significantly increased during the 8-week treatment period, while in CQR-300 group, body fat gradually decreased from T0 to T8. CQR-300 administered for 8 weeks brought about a 12.8% reduction in body fat, which was significantly different (*p* < 0.05) from the change observed in the placebo group (Table 2b).

**Table 2b.** Effect of CQR-300 on Percentage Body Fat Measured by DEXA

	*T0 (%)*	*T2 (%)*	*T4 (%)*	*T8 (%)*	*T0–T2*	*T0–T4*	*T0–T8*	*% change after 8 weeks*
Placebo	44.1 ± 8	44.5 ± 4	44.1 ± 7	44.2 ± 4.5	0.3 ± 1	0.2 ± 0.5	−0.1 ± 0.5	+0.3 ± 4.5
CQR-300	45.3 ± 7.12	45.0 ± 8.21^a^	42.3 ± 4.92^*a^	39.5 ± 8.76^*a^	0.3 ± 1.0	5.1 ± 2.1^*^	7.9 ± 2.1^*^	−12.8 ± 6.0^*^

Values are means ± SD. Statistical significance is at ^*^p ≤ 0.05, for comparison of differences between placebo and the CQR-300 group at each time point or ^a^*p* ≤ 0.05, for comparing of differences compared to T_0_ within the same group.

CQR-300, 300 mg of *Cissus quandrangularis*; DEXA, dual-energy X-ray absorptiometry.

#### Waist circumference

CQR-300 intake over the 8 weeks experimental period, brought about a 8.9% decrease in waist circumference compared to placebo (1.6%) (Table 3a).

**Table 3a.** Effect of CQR-300 on Waist Circumference (cm)

	*T0 (cm)*	*T8 (cm)*	*T0–T8*	*% decrease after 8 weeks*
Placebo	94.5 ± 8.0	93.2 ± 7.0	1.6 ± 1.0	1.6 ± 0.5
CQR-300	95.8 ± 12.04	86.9 ± 11.50^*a^	8.6 ± 2.7^*^	8.9 ± 2.1^*^

Values are means ± SD. Statistical significance is at ^*^p ≤ 0.05, for comparison of differences between placebo and the CQR-300 group at each time point or ^a^*p* ≤ 0.05, for comparing of differences compared to T_0_ within the same group.

CQR-300, 300 mg of *Cissus quandrangularis*.

#### Hip circumference

An 8 weeks treatment led to a decrease in hip circumference in both groups. The reduction was more marked (*p* < 0.05) in CQR groups than in the placebo group (Table 3b).

**Table 3b.** Effect of CQR-300 on Hip Circumference (cm)

	*T0 (cm)*	*T8 (cm)*	*T0–T8*	*% decrease after 8 weeks*
Placebo	117.6 ± 5.5	115.4 ± 5.0	2.1 ± 2.0	1.8 ± 2.0
CQR-300	118.5 ± 5.47	109.4 ± 6.02^*a^	9.1 ± 2.7^*a^	7.7 ± 2.1^*^

Values are means ± SD. Statistical significance is at ^*^p ≤ 0.05, for comparison of differences between placebo and the CQR-300 group at each time point or ^a^*p* ≤ 0.05, for comparing of differences compared to T_0_ within the same group.

CQR-300, 300 mg of *Cissus quandrangularis*.

#### Waist to Hip ratio

The waist to hip ratio was significantly (*p* < 0.05) reduced in the CQR-300 group over the 8-week experimental period (Table 3c).

**Table 3c.** Effect of CQR-300 on Waist to Hip Ratio

	*T0*	*T8*	*T0–T8*	*% change after 8 weeks*
Placebo	0.80 ± 0.4	0.81 ± 0.6	−0.01 ± 0.05	+1.25 ± 0.05
CQR-300	0.81 ± 0.3	0.79 ± 0.54	0.02 ± 0. 41	−2.50 ± 0.41^*^

Values are means ± SD. Statistical significance is at ^*^p ≤ 0.05, for comparison of differences between placebo and the CQR-300 group.

CQR-300, 300 mg of *Cissus quandrangularis*.

#### Systolic blood pressure

An 8 weeks treatment with CQR-300 reduced (*p* < 0.05) systolic blood pressure by 8.7% compared with the placebo group (0.30%) (Table 4a).

**Table 4a.** Effect of CQR-300 on Systolic Blood Pressure

	*T0*	*T8*	*T0–T8*	*% decrease after 8 weeks*
Placebo	127.6 ± 5.5	126.2 ± 5.5	1.4 ± 0.5	0.3 ± 0.5
CQR-300	133.2 ± 4.38	121.6 ± 5.47^*a^	11.6 ± 3.2^*a^	8.7 ± 2.7^*^

Values are means ± SD. Statistical significance is at ^*^p ≤ 0.05, for comparison of differences between placebo and the CQR-300 group at each time point or ^a^*p* ≤ 0.05, for comparing of differences compared to T_0_ within the same group.

CQR-300, 300 mg of *Cissus quandrangularis*.

#### Diastolic blood pressure

There was a decrease in diastolic blood pressure after 8 weeks of treatment in both placebo and CQR-300 groups. However, the reduction was significantly (*p* < 0.05) greater in the CQR-300 group (6.0%) compared with the placebo group (3.29%) (Table 4b).

**Table 4b.** Effect of CQR-300 on Diastolic Blood Pressure

	*T0*	*T8*	*T0–T8*	*% decrease after 8 weeks*
Placebo	98.2 ± 6.5	98.3 ± 5.5	0.1 ± 0.5	3.3 ± 0.5
CQR-300	96.4 ± 6.02	92.3 ± 6.02^*^	4.1 ± 2.0^*^	6.0 ± 3.2^*^

Values are means ± SD. Statistical significance is at ^*^p ≤ 0.05, for comparison of differences between placebo and the CQR-300 group.

CQR-300, 300 mg of *Cissus quandrangularis*.

#### Total cholesterol

There was no difference in total cholesterol levels of placebo and CQR-300 at baseline (T0). Total cholesterol levels reduced from 197.52 to 192.63 mg/dL in the placebo group after 8 weeks treatment. CQR-300, on the contrary, reduced blood cholesterol from 195.77 to 177.02 mg/dL. There was a significant (*p* < 0.05) difference between the CQR-300 and the placebo group after 8 weeks of treatment (Table 5a).

**Table 5a.** Effect of CQR-300 on Plasma Total Cholesterol (mg/dl)

	*T0*	*T8*	*T0–T8*	*% decrease after 8 weeks*
Placebo	197.52 ± 5.1	192.63 ± 4.95	4.88 ± 0.8	2.46 ± 0.35
CQR-300	195.77 ± 5.1	177.02 ± 5.7^*a^	18.74 ± 2.24^*^	9.58 ± 1.1^*^

Values are means ± SD. Statistical significance is at ^*^p ≤ 0.05, for comparison of differences between placebo and the CQR-300 group at each time point or ^a^*p* ≤ 0.05, for comparing of differences compared to T_0_ within the same group.

CQR-300, 300 mg of *Cissus quandrangularis*.

#### Triglycerides

After 8 weeks treatment with CQR-300, plasma triglycerides reduced significantly (*p* < 0.05) by 9.40% compared with an increase in placebo group from 137.43 to 139.31 mg/dL (Table 5b).

**Table 5b.** Effect of CQR-300 on Plasma Triglycerides (mg/dl)

	*T0*	*T8*	*T0–T8*	*% change after 8 weeks*
Placebo	137.43 ± 5.35	139.31 ± 6.16	−1.88 ± 1.0	+1.36 ± 0.8
CQR-300	139.13 ± 5.9	126.03 ± 11.44^*a^	13.14 ± 5.3^*^	−9.40 ± 3.1^*^

Values are means ± SD. Statistical significance is at ^*^p ≤ 0.05, for comparison of differences between placebo and the CQR-300 group at each time point or ^a^*p* ≤ 0.05, for comparing of differences compared to T_0_ within the same group.

CQR-300, 300 mg of *Cissus quandrangularis*.

#### LDL-cholesterol

The administration of CQR-300 to overweight participants significantly (*p* < 0.05) decreased LDL-cholesterol levels by 17.70% over the 8-week period. During this time, the LDL-cholesterol in the placebo group was decreased by 5.48% (127.61–120.62 mg/dL) (Table 5c).

**Table 5c.** Effect of CQR-300 on Plasma LDL Cholesterol (mg/dL)

	*T0*	*T8*	*T0–T8*	*% change after 8 weeks*
Placebo	127.61 ± 8.26	120.62 ± 9.2	6.95 ± 5.65	5.48 ± 2.1
CQR-300	127.21 ± 8.46	104.67 ± 7.44^*a^	22.53 ± 5.7^*^	17.70 ± 3.4^*^

Values are means ± SD. Statistical significance is at ^*^p ≤ 0.05, for comparison of differences between placebo and the CQR-300 group at each time point or ^a^*p* ≤ 0.05, for comparing of differences compared to T_0_ within the same group.

CQR-300, 300 mg of *Cissus quandrangularis*.

#### HDL-cholesterol

An 8-week administration of CQR-300 to overweight participants significantly (*p* < 0.05) increased HDL-c levels by 15.71% compared with a 4.36% increase in the placebo group from 42.42 to 44.15 mg/dL (Table 6).

**Table 6.** Effect of CQR-300 on Plasma HDL Cholesterol (mg/dl)

	*T0*	*T8*	*T0–T8*	*% increase after 8 weeks*
Placebo	42.42 ± 5.25	44.15 ± 4.5	−1.73 ± 0.8	4.36 ± 2.5
CQR-300	40.73 ± 8.9	47.13 ± 5.53^*a^	−6.40 ± 1.9^*^	15.71 ± 3.6^*^

Values are means ± SD. Statistical significance is at ^*^p ≤ 0.05, for comparison of differences between placebo and the CQR-300 group at each time point or ^a^*p* ≤ 0.05, for comparing of differences compared to T_0_ within the same group.

CQR-300, 300 mg of *Cissus quandrangularis*.

#### Fasting blood glucose

CQR-300 significantly (*p* < 0.05) reduced the fasting blood glucose level by 25.27% (4.59–1.08 mmol/L) after 8 weeks compared with a 3.79% decrease in the placebo, from 4.76 to 4.68 mmol/L (Table 7).

**Table 7.** Effect of CQR-300 on Fasting Blood Glucose (mmol/L)

	*T0*	*T8*	*T0–T8*	*% change after 8 weeks*
Placebo	4.76 ± 1.5	4.68 ± 1.5	0.16 ± 0.00	3.79 ± 1.3
CQR-300	4.59 ± 1.31	3.50 ± 2.08^*a^	1.08 ± 0.05^*^	25.27 ± 6.1^*^

Values are means ± SD. Statistical significance is at ^*^p ≤ 0.05, for comparison of differences between placebo and the CQR-300 group at each time point or ^a^*p* ≤ 0.05, for comparing of differences compared to T_0_ within the same group.

CQR-300, 300 mg of *Cissus quandrangularis*.

#### Leptin and adiponectin

Serum leptin level in the CQR-300 group was significantly (*p* < 0.05) reduced (27.38–19.46 mg/dL), while adiponectin levels were significantly (*p* < 0.05) increased (18.34–28.76 mcg/dL) over the 8-week experimental period. In this period, only slight changes in leptin and adiponectin (28.78–26.42 mg/dL and 16.64–17.26 mcg/dL, respectively) were observed in the placebo group (Table 8).

**Table 8.** Effect of CQR-300 on Leptin and Adiponectin Levels

	*Leptin (mg/dL)*				*Adiponectin (mcg/dL)*			
	*T0*	*T8*	*T0–T8*	*% change after 8 weeks*	*T0*	*T8*	*T0–T8*	*% change after 8 weeks*
Placebo	28.78 ± 5.6	26.42 ± 15.6	2.36 ± 2.2	8.2 ± 4.3	16.64 ± 7.75	17.26 ± 10.8	−0.62 ± 1.6	3.5 ± 3.1
CQR-300	27.38 ± 12.6	19.46 ± 11.0^*a^	7.92 ± 2.4^*^	28.9 ± 5.3^*^	18.34 ± 9.02	28.76 ± 12.92^*a^	−10.42 ± 1.4^*^	36.2 ± 6.2^*^

Values are means ± SD. Statistical significance is at ^*^p ≤ 0.05, for comparison of differences between placebo and the CQR-300 group at each time point or ^a^*p* ≤ 0.05, for comparing of differences compared to T_0_ within the same group.

CQR-300, 300 mg of *Cissus quandrangularis*.

## Discussion

The aims of this study were to confirm the ability of CQR-300 to improve components of MS, as well as to validate the changes in body fat using the DEXA method.

Over an 8-week period, the daily use of 300 mg of CQR by overweight participants significantly reduced their weight, body fat, waist and hip circumferences, blood pressure, total and LDL cholesterol, triglycerides, glucose, and leptin concentrations. This was accompanied by increases in the HDL cholesterol and adiponectin concentrations, which could be through mechanisms similar to that observed in experiments with grape seeds.^30^

The reduction of body fat observed over the 8-week trial period was determined using the TANITA bioelectrical impedance meter as well as the DEXA method. The values for body fat as determined by DEXA were higher than values obtained using BIA. This might represent a greater sensitivity of the DEXA measurements (Tables 2a, b).

The overweight condition is characterized by a modification involving the accumulation of fat or triacylglycerol in the adipose tissue through hyperplasia and hypertrophy, which finally leads to an increase in body weight through excessive energy intake and storage. This condition which is linked to perturbations in lipid and carbohydrate metabolism is a major public health concern that requires attention. The loss of weight observed in this pilot study was comparable to that observed in a previous *C. quadrangularis* study, sibutramine administration for 1 year, or the use of orlistat for 6 months or 1 year.^17,31,32^ This weight reduction could be linked to the ability of *C. quadrangularis* to reduce appetite.^33^ In the present study, the CQR-300 linked decrease in body weight as well as the improvement in the different parameters over a relatively shorter period of time could be beneficial in the prevention and management of metabolic disease.

The reduction in fat (Tables 3a, b) was consistent with the reduction of the hip and waist circumferences, which are often used as surrogates in the determination of body fat. These measures have been reported to have moderate correlations with the absolute and relative amounts of visceral adipose tissue as determined by imaging techniques such as DEXA.^34^ Given the controversy on the use of BIA for epidemiologic and clinical/follow-up trials,^23,35,36^ the present study shows that CQR-300 decreased body fat by 8.9% and 12.8% as measured by impedance and DEXA, respectively (Tables 2a, b). It also indicates that the DEXA measures adipose tissue mass and distribution with greater accuracy.^37^ These findings are in accordance with works previously carried out on *Cissus quadrangularis*.^15,17^ Blood pressure, another component of MS was reduced in overweight participants after 8 weeks of treatment. It is well established that weight gain is associated with elevated blood pressure and that subsequent reduction in weight brings about a reduction in BP.^38,39^ It appears that CQR-300 in reducing BP may be a better alternative to multidrug regimens often required by hypertensive patients.^40^

## Conclusions

In this pilot study, the aqueous extract of leaves and stems of *C. quadrangularis*, CQR-300, administered as a single 300 mg dose daily over an 8-week period reduced body fat as demonstrated by both bioelectrical impedance and DEXA measurements. This was paralleled by a reduction in body weight as well as the improvement of certain biochemical parameters associated with MS.

## References

[1] Haslam DW, James WP Obesity. Lancet 2005;366:197–209 16198769 10.1016/S0140-6736(05)67483-1

[2] WHO fact sheet Obesity and overweight. Online document at: www.who.int/mediacentre/factsheets/fs311/en/, accessed 211, 2018

[3] Howard BV, Ruotolo G, Robbins DC Obesity and dyslipidemia. Endocrinol Metab Clin North Am 2003;32:855–867 14711065 10.1016/s0889-8529(03)00073-2

[4] Rinaldi AE, de Oliveira EP, Moreto F, et al. Dietary intake and blood lipid profile in overweight and obese schoolchildren. BMC Res Notes 2012;5:598 23111146 10.1186/1756-0500-5-598 PMC3532384

[5] Bakris GL, Sowers JR ASH position paper: Treatment of hypertension in patients with diabetes - an update. J Clin Hypertension 2008;10:707–713 10.1111/j.1751-7176.2008.00012.x PMC8673265 18844766

[6] Aggoun Y Obesity, metabolic syndrome and cardiovascular disease. Pediatr Res 2007;61:653–659 17426660 10.1203/pdr.0b013e31805d8a8c

[7] Stranahan AM, Lee K, Pistell PJ, et al. Accelerated cognitive aging in diabetic rats prevented by lowering corticosterone levels. Neurobiol Learn Mem 2008;90:479–483 18579418 10.1016/j.nlm.2008.05.005 PMC2600483

[8] Slavin JL Dietary fiber and body weight. Nutrition 2005;21:411–418 15797686 10.1016/j.nut.2004.08.018

[9] Koh-Banerjee P, Rimm EB Whole grain consumption and weight gain: A review of the epidemiological evidence, potential mechanisms and opportunities for future research. Proc Nutr Soc 2003;62:25–29 12740053 10.1079/PNS2002232

[10] Anderson JW, Allgood LD, Lawrence A, et al. Cholesterol-lowering effects of psyllium intake adjunctive to diet therapy in men and women with hypercholesterolemia: Meta-analysis of 8 controlled trials. Am J Clin Nutr 2000;71:472–479 10648260 10.1093/ajcn/71.2.472

[11] Czernichow S, Vergnaud A, Galan P, et al. Effects of long-term antioxidant supplementation and association of serum antioxidant concentrations with risk of metabolic syndrome in adults. Am J Clin Nutr 2009;90:329–335 19491388 10.3945/ajcn.2009.27635

[12] Ghouse MS A Pharmacognostical Review on *Cissus quadrangularis* Linn. Int J Res Pharm Biosci 2015;2:28–35

[13] Sharp H, Hollinshead J, Bartholomew BB, et al. Inhibitory Effects of *Cissus quadrangularis* L. Derived components on lipase, amylase and α-glucosidase activity in vitro. Nat Prod Commun 2007;2:817–822

[14] Kothari SC, Shivarudraiah P, Venkataramaiah SB, et al. Safety assessment of *Cissus quadrangularis* extract (CQR-300): Subchronic toxicity and mutagenicity studies. Food Chem Toxicol 2011;49:3343–3357 21983486 10.1016/j.fct.2011.09.029

[15] Oben J, Kuate D, Agbor GA, et al. The use of a *Cissus quadrangularis* formulation in the management of weight loss and metabolic syndrome. Lipids Health Dis 2006;5:24 16948861 10.1186/1476-511X-5-24 PMC1570348

[16] Oben J, Mandob D, Fomekong GI, et al. The effect of *Cissus quadrangularis* (CQR-300) and a Cissus formulation (CORE) on obesity and obesity-induced oxidative stress. Lipids Health Dis 2007;6:4 17274828 10.1186/1476-511X-6-4 PMC1800848

[17] Kuate D, Nash RJ, Bartholomew B, Penkova Y The use of *Cissus quadrangularis* (CQR-300) in the management of components of metabolic syndrome in overweight and obese participants. Nat Prod Commun 2015;10:1281–1286 26411031

[18] Oben JE, Ngondi JL, Momo CN, et al. The use of a *Cissus quadrangularis/Irvingia gabonensis* combination in the management of weight loss: A double-blind placebo-controlled study. Lipids Health Dis 2008;7:12 18377661 10.1186/1476-511X-7-12 PMC2330043

[19] Lee HJ, Le B, Lee DR, et al. *Cissus quadrangularis* extract (CQR-300) inhibits lipid accumulation by down regulating adipogenesis and lipogenesis in 3T3-L1 cells. Toxicol Rep 2018 [Epub ahead of print]; DOI.org/10.1016/j.toxrep.2018.02.008 10.1016/j.toxrep.2018.02.008 PMC5977379 29854631

[20] Lee HJ, Dong-Ryung, Choi B-K, et al. *Cissus quadrangularis* extracts decreases body fat through regulation of fatty acid synthesis in high-fat diet-induced obese mice. J Appl Biol Chem 2016;59:49–56

[21] Feingold KR, Grunfeld C (2015). Introduction to Lipids and Lipoproteins. In: De Groot LJ, Chrousos G, Dungan K, Feingold KR, Grossman A, Hershman JM, Koch C, Korbonits M, McLachlan R, New M, Purnell J, Rebar R, Singer F, Vinik A, eds. Endotext. South Dartmouth, MA: MDText.com, Inc., 2000–2015

[22] Yi-Chun Li, Chia-Ing Li, Lin Wen-Yuan, et al. Percentage of body fat assessment using bioelectrical impedance analysis and dual-energy X-ray absorptiometry in a weight loss program for obese or overweight chinese adults. PLoS One 2013;8:58272 10.1371/journal.pone.0058272 PMC3613423 23573189

[23] Erceg DN, Dieli-Conwright CM, Rossuello AE, et al. The Stay healthy bioelectrical impedance analyzer predicts body fat in children and adults. Nutr Res 2010;30:297–304 20579521 10.1016/j.nutres.2010.04.009

[24] Eisenkolbl J, Kartasurya M, Widhalm K Underestimation of percentage fat mass measured by bioelectrical impedance analysis compared to dual energy X-ray absorptiometry method in obese children. Eur J Clin Nutr 2001;55:423–429 11423918 10.1038/sj.ejcn.1601184

[25] Staiger H, Tschritter O, Machann JT, et al. Relationship of serum adiponectin and leptin concentrations with body fat distribution in humans. Obes Res 2003;11:368–372 12634431 10.1038/oby.2003.48

[26] Fossati P, Prencipe L Serum triglycerides determined colorimetrically with an enzyme that produces hydrogen peroxide. Clin Chem 1982;28:2077–2080 6812986

[27] Richmond W Preparation and properties of a cholesterol oxidase from Nocardia sp. and its application to the enzymatic assay of total cholesterol in serum. Clin Chem 1973;19:1350–1356 4757363

[28] Friedewald WT, Levy RI, Fredrickson DS Estimation of the concentration of low-density lipoprotein cholesterol in plasma, without use of the preparative ultracentrifuge. Clin Chem 1972;18:499–502 4337382

[29] Trinder P Determination of Glucose in Blood using Glucose Oxidase with an alternative Oxygen acceptor. Ann Clin Biochem 1969;6:24–27

[30] Meeprom A, Sompong W, Suwannphet W, et al. Grape seed extract supplementation prevents high-fructose diet-induced insulin resistance in rats by improving insulin and adiponectin signaling pathways. Br J Nutr 2011;106:1173–1181 21736810 10.1017/S0007114511001589

[31] Smith IG, Goulder MA Randomized placebo-controlled trial of long-term treatment with sibutramine in mild to moderate obesity. J Fam Pract 2001;50:505–512 11407998

[32] Yesilbursa D, Serdar Z, Serdar A, et al. Lipid peroxides in obese patients and effects of weight loss with orlistat on lipid peroxides levels. Int J Obes 2005;29:142–145 10.1038/sj.ijo.0802794 15467775

[33] Yimam M, Jiao P, Hong M, et al. Evaluation of natural product compositions for appetite suppression. J Diet Suppl 2018;14:1–19 10.1080/19390211.2018.1429518 29443598

[34] Verduin WM, Den Helder RV, Doodeman H, et al. Dexa body composition assessment in 10–11 year healthy children. PLoS One 2016;11:e0165275 27788168 10.1371/journal.pone.0165275 PMC5082851

[35] Yi-Chun L, Chia-Ing L, Lin WY, et al. Percentage of body fat assessment using bioelectrical impedance analysis and dual-energy X-ray absorptiometry in a weight loss program for obese or overweight Chinese adults. PLoS One 2012;8:e58272 10.1371/journal.pone.0058272 PMC3613423 23573189

[36] Větrovská RZ Vilikus J, Klaschka Z, et al. Does impedance measure a functional state of the body fat? Physiol Res 2014;63 (Suppl. 2):s309–s320 24908237 10.33549/physiolres.932816

[37] Clasey JL, Hartman ML, Kanaley J, et al. Body composition by DEXA in older adults: Accuracy and influence of scan mode. Med Sci Sports Exerc 1997;29:560–567 9107641 10.1097/00005768-199704000-00020

[38] Azantsa BG, Ntentié R, Mbong AM, et al. Body mass index, blood pressure and hypertension subtypes among untreated hypertensive cameroonians. Br J Med Med Res 2013;3:2231

[39] Millstein RA Measuring outcomes in adult weight loss studies that include diet and physical activity: A systematic review. J Nutr Metab 2014;ID 421423:13 10.1155/2014/421423 PMC4262752 25525513

[40] Duarte JD, Cooper-DeHoff RM Mechanisms for blood pressure lowering and metabolic effects of thiazide and thiazide-like diuretics. Expert Rev Cardiovasc Ther 2010;8:793–802 20528637 10.1586/erc.10.27 PMC2904515

